# Supplementary material for: Efficacy of Combined Use of Everolimus and Second-Generation Pan-EGRF Inhibitors in KRAS Mutant Non-Small Cell Lung Cancer Cell Lines
Source: Int J Mol Sci. 2022 Jul 14;23(14):7774. doi: 10.3390/ijms23147774 (PMC9317664; doi:10.3390/ijms23147774)
Supplement: Supplementary file 1 [file ijms-23-07774-s001.zip › Suplementary tables_150522.pdf]

Efficacy of combined use of everolimus and second-generation pan-EGFR inhibitors in *KRAS* mutant non-small cell lung cancer cell lines

Renato José da Silva-Oliveira[1*]; Izabela Natalia Faria Gomes[1]; Luciane Sussuchi da Silva[1]; André van Helvoort Lengert[1]; Ana Carolina Laus[1]; Matias Eliseo Melendez[1]; Carla Carolina Munari [1]; Fernanda de Paula Cury[1]; Giovanna Barbarini Longato[1]; Rui M Reis[1,2,3*]

[1]Oncology Research Center, Barretos Cancer Hospital, Barretos, São Paulo, Brazil; [2]Life and Health Sciences Research Institute (ICVS) Medical School, University of Minho, Braga, Portugal; [3]ICVS/3B's-PT Government Associate Laboratory, Braga/Guimarães, Portugal.

Correspondence: * Renato J. Silva-Oliveira.
Antenor Duarte Villela, 1331, Zip Code: 14784 400, Barretos, São Paulo, Brazil
Phone/Fax: +55 173 321 6600
E-mail: renatokjso@gmail.com
And
Rui Manuel Reis,
Antenor Duarte Villela, 1331, Zip Code: 14784 400, Barretos, São Paulo, Brazil
Phone/Fax: +55 173 321 6600
E-mail: ruireis.hcb@gmail.com

Table S1 Lung cancer cell lines histological classification and origin.

Cell line	Disease	Tissue	Origin
NCI-H1975	Adenocarcinoma	Lung	BCRJ
NCI-H827	Adenocarcinoma	Lung	BCRJ
PC9	Adenocarcinoma	Lung	BCRJ
SK-MES-1	Squamous Cell Carcinoma	Lung	ECACC
SK-LU-1	Adenocarcinoma	Lung	ECACC
A549	Adenocarcinoma	Lung	ECACC
NCI-H292	Carcinoma; Mucoepidermoid Pulmonary	Lung	BCRJ
COR-L23	Lung large cell carcinoma	Lung	ECACC
COR-L105	Adenocarcinoma	Lung	ECACC
LUDLU-1	Squamous cell carcinoma	Lung	ECACC
NCI-H322	Bronchioalveolar carcinoma	Lung	ECACC
NCI-H358	Adenocarcinoma	Lung	ECACC
NCI-H727	Non-small cell carcinoma	Lung	ECACC
NCI-H2228	Adenocarcinoma	Lung	BCRJ
Calu-3	Adenocarcinoma	Lung	BCRJ

Abbreviations: BCRJ, Rio de Janeiro Cell Bank; ECACC, European Collection of Authenticated Cell Cultures.

Table S2 Genes analyzed by real time PCR.

	Gene	Primer Foward (5' - 3')	Primer Reverse (5' - 3')	Size (bp)	Annealing temperature °C
**Genes EMT/Migration/ Invasion**	β-actin	GGACTTCGAGCAAGAGATGG	AGCACTGTGTTGGCGTACAG	234	63
	Plakoglobin	AAGGTGCTATCCGTGTGTCC	GTTGTTGCATGTCAGGTTGG	261	63
	Snail	CTCTAGGCCCTGGCTGCTAC	TGACATCTGAGTGGGTCTGG	134	63
	Slug	CTTTTTCTTGCCCTCACTGC	ACAGCAGCCAGATTCCTCAT	161	63
	E-cadherin	TGCCCAGAAAATGAAAAAGG	GTGTATGTGGCAATGCGTTC	200	61
	N-cadherin	ACAGTGGCCACCTACAAAGG	TGATCCCTCAGGAACTGTCC	392	64
	Vimentin	GGGACCTCTACGAGGAGGAG	AAGATTGCAGGGTGTTTTCG	177	63
	Fibronectin	TCGAGGAGGAAATTCCAATG	CTCTTCATGACGCTTGTGGA	382	61
	Nanog	ATACCTCAGCCTCCAGCAGA	CTGGGGTAGGTAGGTGCTGA	174	59
	ITGA11a	ATCTCGCAGTCAGCAAACCT	AAGAGGACGTCAGCCTCGTA	305	61
	MMP2	CAGGGAATGAGTACTGGGTCTATT	ACTCCAGTTAAAGGCAGCATCTAC	119	59
	MMP9	GCACGACGTCTTCCAGTACC	CAGGATGTCATAGGTCACGTAGC	124	59
	MMP 14	CACTGCCTACGAGAGGAAGG	TCCCTTCCCAGACTTTGATG	269	63
	MMP 24	TGAAGGCATTGACACAGCTC	CGCTCAGTTTCTGGTTGTCA	242	63

Abbreviations: EMC, Epithelial-mesenchymal transition; BP, base pair

Table S3 Growth inhibition analyses of NSCLC cell lines

Cell line	Allitinib (GI % ± DP)	GI classification score	Afatinib (GI % ± DP)	GI classification score
NCI-H1975	81.9 ± 1.54	HS	52.3 ± 7.32	MS
NCI-H827	89.9 ± 0.79	HS	79.5 ± 2.07	HS
PC9	86.6 ± 0.38	HS	48.3 ± 1.21	MS
SK-MES-1	40.0 ± 3.5	MS	29.0 ± 2.23	R
SK-LU-1	33.7 ± 4.1	R	14.0 ± 1.02	R
A549	9.7 ± 6.8	R	1.0 ± 0.20	R
H292	70.33 ± 1.56	HS	16.0 ± 2.00	R
COR-L23	20.0 ± 2.56	R	2.0 ± 1.78	R
COR-L105	77.3 ± 1.15	HS	72.0 ± 2.64	HS
LUDLU-1	84.0 ± 4.35	HS	34.6 ± 2.51	R

NCI-H322	38.3 ± 3.05	R	50.6 ± 2.08	MS
NCI-H358	54.6 ± 4.50	MS	47.0 ± 4.02	MS
NCI-H727	33.3 ± 3.78	R	54.6 ± 5.03	MS
NCI-H2228	70.5 ± 2.12	HS	18.3 ± 1.08	R
Calu-3	45.1 ± 2.81	MS	35.5	R

Abbreviations: HS, highly sensitive; MS, moderately sensitive; R, resistant.

Supplementary Table S4 Differentially expressed genes involved in the focal adhesion-PI3K-Akt-mTOR-signaling

**Common gene**	**H292 - *KRAS* - G12D**		**H292 - *KRAS* - G12S**	
	Fold Change	*p-value*	Fold Change	*p-value*
SPP1	10.4	0.0000025	32.7	0.000000795
EFNA2	5.0	0.015589	3.3	0.044972
COL11A2	4.6	0.010355	2.9	0.046993
COL3A1	5.2	0.001927	2.8	0.006103
GHR	3.5	0.000953	1.9	0.038975
CREB3L1	1.6	0.0151	3.6	0.004881
ITGB8	8.4	0.0000777	1.7	0.025268
PIK3CG	-2.4	0.000725	-1.7	0.008205
MET	-1.7	0.00841	-1.8	0.01098
IFNA7	-1.8	0.046668	-2.1	0.014226
ITGA6	-2.5	0.011492	-2.2	0.025567
LAMB3	-4.3	0.0000967	-2.3	0.002314
IL2RB	-2.6	0.03335	-2.5	0.02391
ITGB3	-2.2	0.002332	-2.5	0.003119
FGF21	-2.9	0.040006	-2.9	0.026253
TNC	-5.1	0.006592	-4.8	0.008261
THBS1	-16.7	0.000334	-7.3	0.002237
ITGB4	-3.7	0.010307	-8.8	0.000882

Supplementary Table S5 Combination index (CI) calculated in H292 *KRAS* mutant and wild type cell lines

Cell line	$IC_{50\ values}$ (μM)		C.I	$IC_{50\ values}$ (μM)		C. I
	**AFA**	**AFA + EVE**		**ALI**	**ALI + EVE**	
H292 - *KRAS* WT	5.3 ± 0.2	4.1 ± 1.1	> 1	2.7 ± 0.9	1.9 ± 1.1	> 1
H292 - *KRAS* G12D	5.8 ± 0.7	3.9 ± 0.5	<1	2.9 ± 1.2	1.0 ± 0.4	<1
H292 - *KRAS* G12S	8.5 ± 0.5	2.9 ± 0.7	<1	4.2 + 1.1	0.9 ± 0.7	<1

Abbreviations: CI, combination index; AFA, afatinib; ALI, allitinib; EVE, everolimus
