# Supplementary material for: Efficacy of Combined Use of Everolimus and Second-Generation Pan-EGRF Inhibitors in KRAS Mutant Non-Small Cell Lung Cancer Cell Lines
Source: Int J Mol Sci. 2022 Jul 14;23(14):7774. doi: 10.3390/ijms23147774 (PMC9317664; doi:10.3390/ijms23147774)
Supplement: Supplementary file 1 [file ijms-23-07774-s001.zip › Supplementary Figures.pdf]

Supplementary Materials

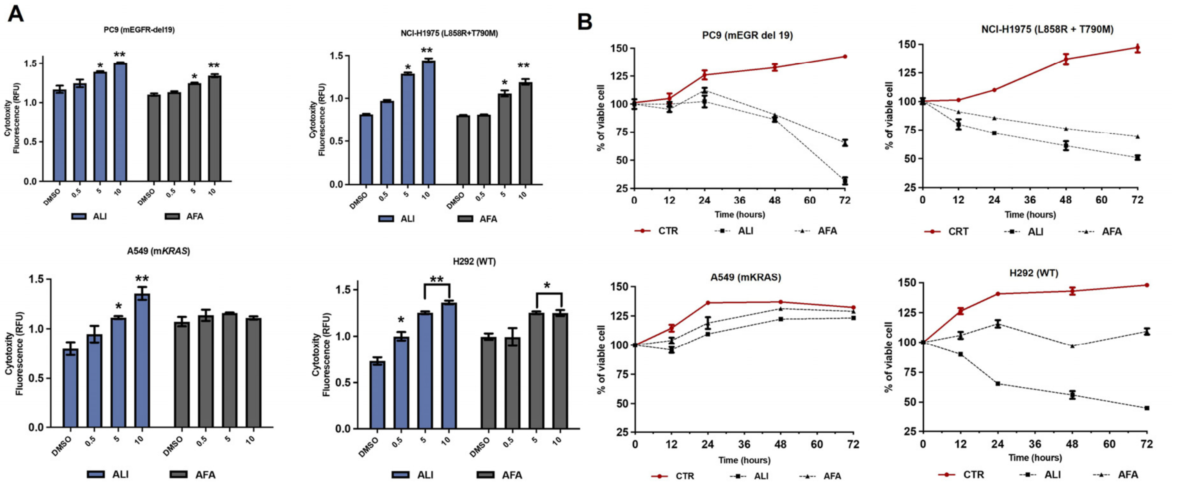

Figure S1. Comparative effect between afatinib and allitinib. (**A**) cytotoxic activity (**B**) cellular proliferation curves. * *p*-values < 0.01, ** *p*-Value < 0.001.

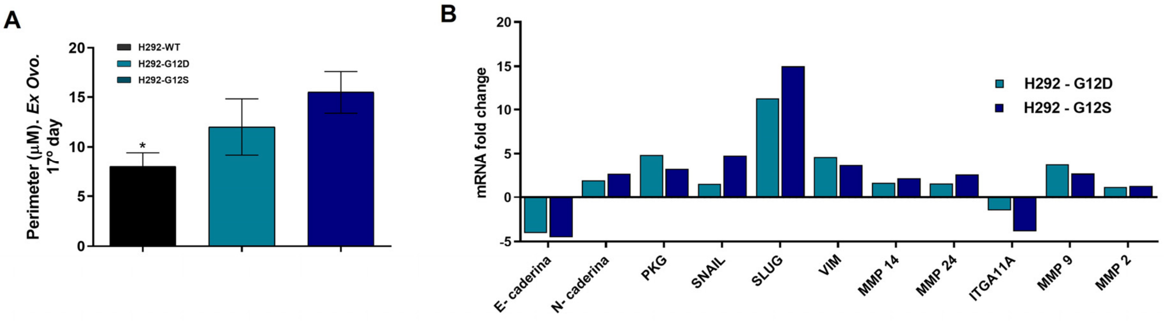

Figure S2. Evaluates chick chorioallantoic membrane (CAM) assay in H292 *KRAS* mutated and wild-type cell lines. (**A**) tumor perimeter (**B**) mesenchymal markers mRNA expression. * *p*-values < 0.01.

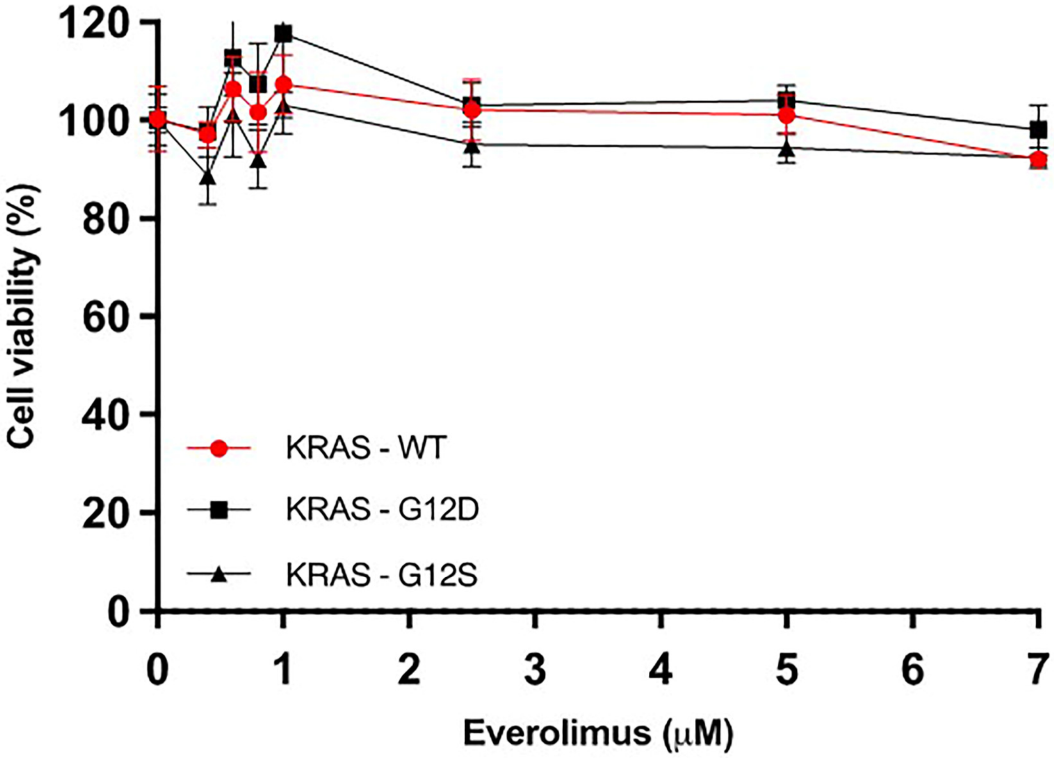

Figure S3. Proliferation curve (MTS).
